# Zirconia abutments and reduced crown-to-implant ratios: Insights into stress distribution

**DOI:** 10.1590/1807-3107bor-2026.vol40.076

**Published:** 2026-10-16

**Authors:** Lorenzo GRASSI, Ayrton Geroncio SILVA, Guilherme Almeida BORGES, Rafael Pino VITTI, Valentim Adelino Ricardo BARÃO, Milton Edson MIRANDA, William Cunha BRANDT, Raphael Cavalcante COSTA

**Affiliations:** (a) São Leopoldo Mandic, Department of Dentistry, Campinas, SP, Brazil.; (b) Universidade Estadual de Campinas – Unicamp, Piracicaba Dental School, Department of Prosthodontics and Periodontology, Piracicaba, SP, Brazil.; (c) Universidade de São Paulo – USP, School of Dentistry, Department of Prosthodontics, São Paulo, SP, Brazil.

**Keywords:** Dental Prosthesis, Finite Element Analysis, Crowns

## Abstract

This study aims to evaluate stress distributions in dental implants, zirconia abutments, and peri-implant bone tissues at different crown-to-implant ratios. In-silico analysis was performed using SolidWorks (SolidWorks Corporation, Concord) to evaluate stress distributions in the implant and abutment based on von Mises stresses, as well as in cortical and trabecular bone under maximum and minimum principal stresses. Implant-supported rehabilitation (4 × 11 mm) was simulated with zirconia abutments measuring 8 mm (RS), 10 mm (SS) and 12 mm (LS), corresponding to crown-to-implant ratios of 0.72:1, 0.90:1 and 1.1:1, respectively. An axial load of 200 N was applied and stress distribution was analyzed using numerical data and color-coded images. The RS group exhibited the highest von Mises stress (188.63 MPa), localized above the first implant thread, while SS and LS showed ~178 MPa. Additionally, RS abutments exhibited higher von Mises stress values in the implant (336.23 MPa, SS and LS: ~325 MPa), concentrated in a smaller area close to the first threads of the implant. Regarding stress in bone tissue, lower values of maximum (1.99 MPa, SS and LS: ~2.4 MPa) and minimum (-8.69 MPa, SS and LS: ~-10.6 MPa) principal stresses were found in the RS group compared to the SS and LS groups, suggesting better performance. No differences in stress distribution values were observed between the SS and LS groups. These findings suggest that a lower crown-to-implant ratio provides the most favorable stress distribution and that abutment cervical-occlusal length influences the effectiveness of oral rehabilitation.

## Introduction

Dental implants are considered the optimum solution for rehabilitating edentulous spaces,^1^ a fact that encourages ongoing research into implant materials and prosthetic abutments to improve functionality and esthetics.^2,3^ Regarding the prosthetic options in rehabilitation with implants, the choice of abutment material is a crucial step since the supporting material plays a pivotal role in the fracture resistance of restorative materials.^4,5^ Additional decisions regarding abutment material and crown-to-implant ratio can also influence biting force or parafunctional habits.^6-8^

Abutment materials can be manufactured from either titanium or zirconia.^9,10^ However, titanium abutments may compromise esthetics, particularly in the anterior region or in posterior rehabilitation, depending on patient-specific factors such as pink-white esthetics and thin soft tissue width, which can lead to the appearance of a metallic line near the peri-implant tissue.^10^ To address this issue, ceramic abutments can be used as an alternative to metallic ones, preventing greyish pigmentation.^11^ Zirconia abutments in particular provide superior esthetics since they better mimic natural teeth and minimize metal shadows, which are more noticeable in thin gingival phenotypes.^9,12^ In addition to esthetics, zirconia abutments promote superior fracture resistance than alumina abutments and reduce bacterial adhesion compared to titanium, making them an excellent choice for oral rehabilitation.^10,13^ Studies^14,15^ suggest that zirconia implants generate less stress in cortical bone. Zirconia abutments also exhibit lower von Mises stresses than titanium abutments, along with reduced compressive stress in cortical bone.^11^ Furthermore, zirconia is highly biocompatible with hard and soft tissues,^6^ further supporting its use in implant dentistry.

The longevity of dental implants is directly affected by mechanical stresses between the alveolar bone and the implant.^16^ Since the intensity and distribution of mechanical strength are crucial for implant survival, the crown-to-implant ratio can influence the distribution of stresses in peri-implant bone tissues.^17,18^ Within this context, von Mises stress analysis provides a single equivalent stress value that accounts for the combined effect of all stress components and is widely used to predict yielding of different materials (e.g., graphene framework, titanium implants, ceramic crowns).^2,11,16,17^ In contrast, principal stress analysis identifies the maximum and minimum normal stresses at a point where shear stresses are zero. These stresses represent the directions in which the material experiences the highest tension (maximum principal stress) and the highest compression (minimum principal stress).^14,15,17^ Moreover, combining variables such as a greater implant-to-bone contact surface and a crown-to-implant ratio of 1:1 has been reported to create a more favorable condition for the longevity of prosthetic rehabilitations.^17,19^ Although an unfavorable crown-to-implant ratio is widely considered potentially harmful, further biomechanical studies are needed to assess alternative ratios (1:1, 1:1.5, 1:1.75, and 1:2) and their impact on biomechanical distribution within implants and their supporting structure.^20^ Consistently, an unfavorable crown-to-implant ratio may pose a risk for prosthetic complications such as screw loosening and abutment fractures.^21^ Furthermore, as crown-to-implant ratios increase, crown height may play a greater role in biomechanical failures.^20^ Conversely, other studies showed that a higher crown-to-implant ratio results in less peri-implant bone loss or that no relationship exists between crown height and technical or biological complications.^22,23^

Despite considerable controversy in the literature, peri-implant issues related to the crown-to-implant ratio may contribute to peri-implant bone loss, underscoring the need for further biomechanical studies to support clinical effectiveness research. Additionally, factors such as the clinician’s experience, available bone for implant placement, location, and prosthetic space for rehabilitation can to some extent influence decisions regarding prosthetic treatment.^24^ However, data on the effects of a reduced crown-to-implant ratio on stress distribution are scarce.

Given the ongoing controversy, this study aims to evaluate stress distributions in implants, zirconia abutments, and peri-implant bone tissues at different crown-to-implant ratios ratios. Specifically, we assessed the impact of a reduced crown-to-implant ratio (0.72:1) compared to a standard ratio (0.90:1) and a large ratio (1.1:1) on stress distribution using 3D finite element analysis. The null hypothesis states that the crown-to-implant ratio does not influence the biomechanical performance of the prosthetic system in distributing stress.

## Methods

In-silico analysis using 3D finite element modeling was performed to evaluate stress distributions in implants, abutments, and bone tissues during simulated rehabilitation of a lower left premolar. The computer-aided design (CAD) structures of each model were created to obtain an external hexagon titanium implant measuring 4 × 11 mm, paired with a zirconia abutment and a lithium disilicate crown. Images of the prosthetic components were obtained from a database. The finite element models were constructed using the SolidWorks software (SolidWorks Corporation, Concord, USA). The different crown-to-implant ratios were the independent variable. As dependent variables, stress distribution was evaluated. Maximum and minimum stresses were assessed for the bone tissue. For the abutment and implant, von Mises stresses were analyzed based on previous studies.^25,26^ Three crown heights were analyzed: 8 mm (reduced size, RS), 10 mm (standard size, SS), and 12 mm (large size, LS), corresponding to different crown-to-implant ratios (RS: 0.72:1; SS: 0.90:1; LS: 1.1:1). Heights were measured as the distance from the implant platform to the site of load application, which was aligned axially along the structure’s axis. Finite element analysis was carried out in three steps: pre-processing (creation of the CAD model), processing (mathematical calculation of the finite element simulation), and post-processing (collection and analysis of the results).^25^ A computational load of 200 N was applied to simulate the functional forces of a partially edentate patient.^26^

In the pre-processing step, the geometric models were constructed individually (implant, cortical and trabecular bone, abutment). For the section of the mandible where the implants are placed, an artificial mandible was used as the basis for constructing the model. To align with the objectives of our study, modifications were made to the anatomy of the generic model, including the removal of all teeth, using the *untrim* and *surface-by-edge* commands. After individual modeling, the structures (cortical bone, cancellous bone, implant, and abutment) were assembled into 3D models to simulate a clinical scenario involving the absence of the lower left first premolar.

In the processing step, relevant data regarding the physical-mechanical properties of the materials used in the construction of the models were added.^27^ For these constructions, the simulation was configured with representative material properties, including Young’s modulus and Poisson’s ratio, and boundary conditions were defined to accurately replicate real clinical conditions. All materials were individually imported into the finite element analysis software (ANSYS Workbench 14.0; Swanson Analysis Inc). Next, the values of Young’s modulus and Poisson’s ratio (Table 1) were inserted for each material. The cortical and trabecular bone tissues were modeled as isotropic, homogeneous, linearly elastic, and completely osseointegrated in the implant. All contacts were considered to be perfectly attached (frictionless). For boundary conditions, rigid fixation was applied to the posterior regions of the cortical and cancellous bone to prevent displacement in the x, y, and z Cartesian planes.

**Table 1:** Mechanical properties of the material.

Material	Young modulus (GPa)	Poisson’s ratio
Cortical bone^18^	13.7	0.30
Trabecular bone^18^	1.37	0.30
Titanium^18^	115	0.33
Zirconia^18^	200	0.31
Lithium disilicate^19^	68	0.24

The structure of the models was divided into a finite number of elements, which were interconnected by nodal points located in the three-dimensional coordinate system. The resulting assembly was referred to as a mesh. The files were saved in step format and reorganized into components to facilitate both visualization and the mesh creation process. As part of the refinement process, the interfaces between solids were optimized using a maximum element size of 0.4 mm, a growth factor of 1.5 for the elements, and 10-node tetrahedral elements.^28,29^ The finite element meshes were then generated and their integrity was assessed in terms of element quality in the regions of interest. A mesh was created on each surface of the solids that constitute the model. To minimize numerical errors and divergences during analysis, meshing was performed sequentially, starting with the innermost part of the assembly and progressing outward. The mesh from each part was copied and used as the base for the next group, ensuring consistency across the model. All 3D meshes were built using at least three elements in each dimension in order to obtain accurate results with a higher degree of convergence.^30^ Occlusal load was applied computationally at an intensity of 200 N, axially along the axis of the structures.^26^ To understand the stress distribution mechanism, mathematical equations (processing analysis) that included von Mises stress values for abutments and implants and maximum and minimum principal stresses for cortical and trabecular bone were created for all models using ANSYS Workbench 14 solver.

For processing analysis, the command analysis was set according to the material. First, the ductile and friable materials were assessed based on the equivalent von Mises stress for implants and abutments.^31^ Since cortical bone is a brittle material (nonductile structure), the maximum and minimum principal stresses were obtained to better understand the influence of different prosthesis designs on peri-implant bone tissues^26^. Next, stresses were plotted numerically, illustrated in color-coded stress maps, and compared among the three groups (RS, SS, and LS). Stress peaks were removed to avoid misleading information in the output, especially in critical areas such as the interface between the implant and abutment and/or between the implant and bone tissue.^31,32^ Statistical analysis was not performed because, once numerical convergence is achieved in finite element modeling, the results become stable, reproducible, and independent of mesh or time parameters, making replication unnecessary.^33^

## Results

The images provided by finite element analysis showed that the RS group with a crown height of 8 mm (Figure 1) exhibited the highest maximum von Mises stress (188.63 MPa) (Table 2) on the zirconia abutment, with concentration in the distal and superior regions along its entire circumference (Figure 2). The SS and LS groups exhibited the same maximum von Mises stress value (177.87 MPa), with a larger stress distribution area compared to the RS group (Figure 1).

**Figure 1:**
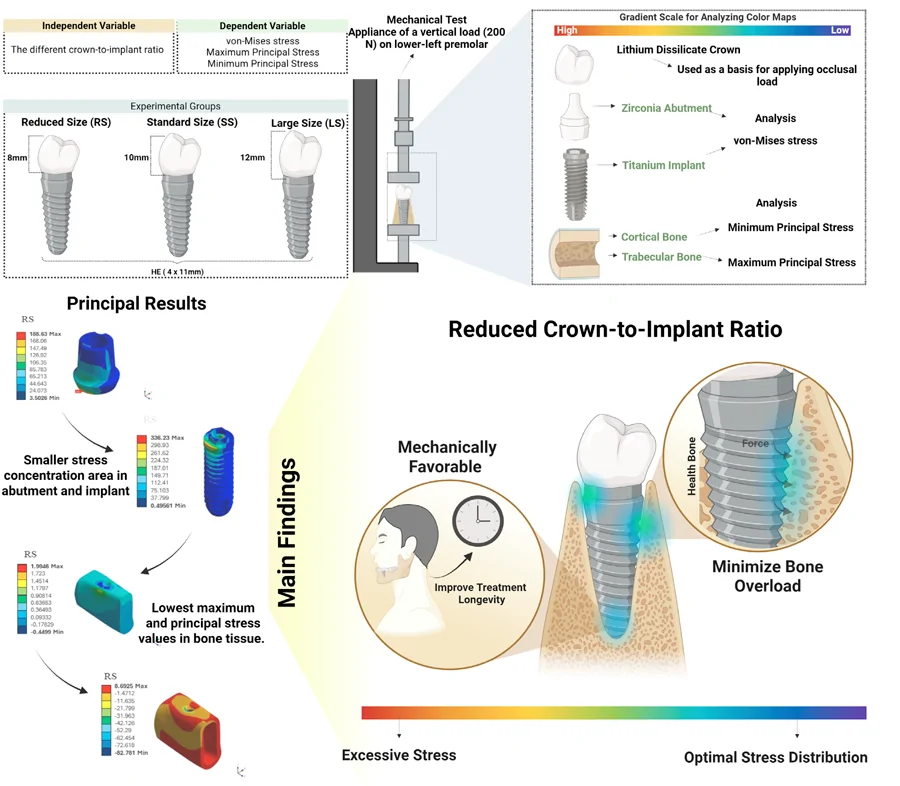
Von Mises stress maps in prosthetic structures, abutments (upper part of the image) and implants (lower part of the image).

RS: reduced size: 0.72:1; SS: standard size: 0.90:1; LS, large size: 1.1:1.

**Table 2:** Von Mises stresses in abutments and implants and maximum and minimum principal stresses in cortical and trabecular bone

Group	Maximum (MPa)	Minimum (MPa)
Von Mises stress (abutment)		
RS	188.63	3.5
SS	177.87	3.39
LS	177.87	3.39
Von Mises stress (implant)		
RS	336.23	0.49
SS	324.55	0.49
LS	324.55	0.49
Minimum principal stress (cortical bone)		
RS	-8.69	-82.78
SS	-10.59	-97.06
LS	-10.59	-97.06
Maximum principal stress (trabecular bone)		
RS	1.99	0.44
SS	2.35	0.47
LS	2.35	0.47

RS, reduced size; SS, standard size; LS, large size.

**Figure 2:**
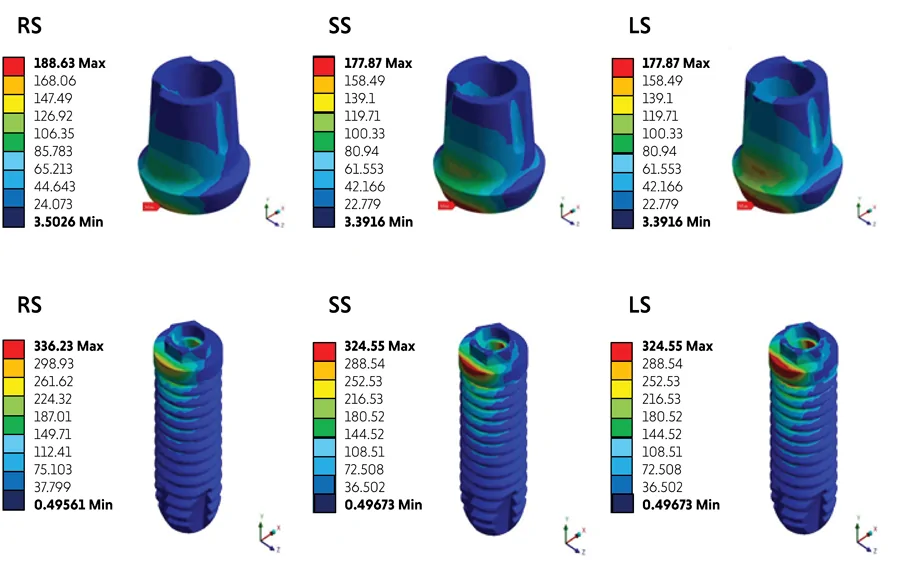
Von Mises stress maps in prosthetic structures, abutments (upper part of the image) and implants (lower part of the image).

RS: reduced size: 0.72:1; SS: standard size: 0.90:1; LS: large size: 1.1:1.

Regarding stress distribution on the implant, the RS group exhibited the highest von Mises stress value (336.23 MPa) (Table 2) in a smaller area, which was located above the first thread of the implant (Figure 2) at the mesial edge in the platform region. The LS and the SS groups showed a larger stress distribution area than the RS group, with identical maximum von Mises stress values (324.55 MPa) in both groups (Table 2). Stress distribution in the area decreased in the last threads of the implant around its entire circumference and along the distal side throughout the implant’s length (Figure 1). Stress distribution was more favorable in the RS group, as demonstrated by a smaller area of stress concentration compared to the other groups. The red-shaded areas, indicative of higher stress and constant fatigue, appear more limited and located near the first implant threads in the RS group. In contrast, as the crown-to-implant ratio increases, these areas expand in the SS and LS groups, suggesting a greater overload on the bone structures surrounding the implant (Figure 1).

Assessment of the effect of different crown-to-implant ratios on cortical bone under compressive stress (Figure 2) revealed that the group with the smallest abutment height (RS) had the lowest minimum principal stress value (-82.78 MPa) (Table 2) in bone tissue. This stress was located in the mesial region, in the upper portion of the ridge near the first threads of the implant (Figure 2). It is important to note that the minimum principal stress did not differ between the SS and LS groups (Table 2), both reaching a value of -97.06 MPa; however, there was a qualitative difference between these groups. The RS group exhibited a lower intensity of compressive stresses compared to the other two groups, suggesting that a shorter crown height may reduce the risk of bone overload.

Regarding the performance of trabecular bone under tensile stresses (Table 2), the RS group obtained the lowest maximum principal stress value (1.99 MPa), located in the mesial region, in the outermost part near the upper portion of the ridge, and in the distal region, in the upper portion of the ridge near the first threads of the implant (Figure 2). There was no quantitative difference in the maximum stress value between the SS and LS groups, both reaching 2.35 MPa (Table 2). Qualitatively, the RS group exhibited the most favorable stress distribution, as demonstrated by larger blue- and green-shaded areas, indicating lower stress concentration throughout the bone structure. In contrast, the SS and LS models displayed more extensive, yellow-shaded regions around the implant-bone interface, suggesting higher stress concentration (Figure 2).

## Discussion

The primary aim of investigating stress distribution in abutments and implants is to provide essential information for implant rehabilitation, considering the gradient of stress in bone areas.^34^ Complementarily, imbalances in masticatory functions can generate an overload in the bone-implant area, causing failure and consequent implant loss.^6^ Von Mises and principal stress analyses are widely recommended for evaluating the mechanical behavior of a material under complex loading conditions.^17,35^ Regarding implant-supported prostheses, an unfavorable crown-to-implant ratio (> 1:1) can lead to biomechanical complications over time, mainly because implants are less resistant to oblique forces.^34,35^ The mechanical properties of dental materials are important for successful and stable osseointegration, while occlusal forces combined with early loading requirements can lead to the failure of implants.^36^ Nevertheless, implant materials with a lower elastic modulus are beneficial since they reduce stress.^36^

Based on our results, the null hypothesis was rejected since the shorter crown (8 mm) showed a better performance in terms of stress distribution and resistance to tensile and compressive forces compared to the other groups. It is important to note that the long implant (>10 mm) was chosen because it can provide greater primary stability and a more favorable distribution of occlusal loads, owing to the increased contact surface between the implant and surrounding bone tissue.^19^The present study revealed that shorter abutments (8 mm), corresponding to the RS group, exhibited the highest maximum von Mises stress value (Table 2) on the zirconia abutment located near the prosthetic interface between the abutment and implant, but within a smaller area compared to the other groups. Although high (188.63 MPa), this value is still considered below the material’s strength, preventing cracks or ruptures.^37,38^ There were no differences between the SS and LS groups; however, the color maps suggest that the stress concentration area increases proportionally with increasing crown-to-implant ratio (Figure 1). The higher Young modulus of zirconia (Table 1) may induce deformation in the material with the lower modulus, in this case, titanium.^39^ However, the performance observed in the RS group may be beneficial because a lower crown-to-implant ratio helps reduce unequal wear between surfaces, even when different materials are used.^39^ Additionally, external hexagonal connections promote better stress distribution when compared to other connection types. Thus, the RS group may be resistant to occlusal forces and the smaller area of stress distribution may decrease microgaps and screw loosening of the abutments, promoting longer survival rates of implant rehabilitations.^34,35^

Regarding stress distribution in the implant, variations in crown and abutment height influenced stress dissipation despite consistent implant dimensions. The RS group showed the highest von Mises stress value (Table 2), while no significant differences were observed between the SS and LS groups. This finding might be explained by the fact that a crown with a lower elastic modulus, such as a lithium disilicate crown, can transfer stress to the cervical region near the first implant thread, the platform, and the bone site^6,40^ (Figure 1). Furthermore, as the crown-to-implant ratio increases, the fatigue area expands, potentially increasing stress in the peri-implant bone and causing complications in the prosthetic system. Contrary to the findings of Nissan et al.,^20^ who suggested that a crown-to-implant ratio greater than 1:1 results in a higher incidence of biomechanical issues, our results indicate that implant length does not affect implant fracture resistance or biomechanical complications. Instead, stress distribution appears to be more influenced by the crown and abutment. In addition, a reduced mechanical lever effect was observed in the RS group, which resulted in a more homogeneous stress distribution in the cervical region as demonstrated by less intense red-shaded areas.

Evaluation of the bone resorption process is valid since excessive load or poor stress distribution during masticatory function can damage the alveolar bone, causing resorption and eventual failure of osseointegration. Analysis of principal stresses in bone tissue revealed that the shortest abutment height (RS) resulted in the lowest maximum and principal stress values (Table 2), while no significant differences in trabecular or cortical bone were observed between the SS and LS groups, whose overall stress values were higher. In addition, the RS group showed better performance in trabecular and cortical bone compared to the SS and LS groups (Figure 3), suggesting that a lower crown-to-implant ratio enhances resistance to compressive and tensile forces, potentially contributing to prolonged marginal bone stability. This performance can be explained by the fact that an increase in the crown-to-implant ratio can concentrate stress on the fixation screw and surrounding bone tissue (Figure 3), causing marginal bone loss and creating a lever-arm effect at the bone crest, which could ultimately compromise rehabilitation.

**Figure 3:**
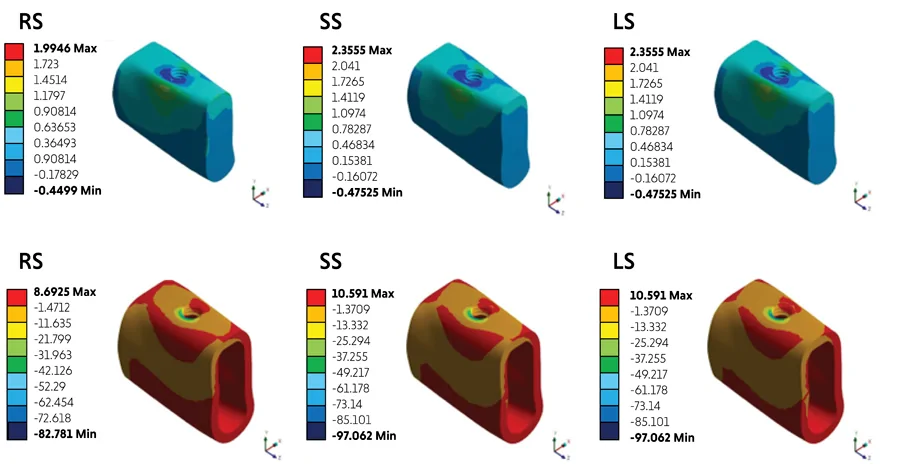
Tensile and compressive stress maps in prosthetic structures, cancellous bone (upper part of the image), and cortical bone (lower part of the image).

RS: reduced size: 0.72:1; SS: standard size: 0.90:1; LS: large size: 1.1:1.

This study has some limitations, including the use of a linear computer simulation, the assumption of bone tissue as homogeneous and linearly elastic, and the absence of oblique loading on the crown. Additionally, the lack of complementary in vitro validation using step-stress accelerated life testing limits extrapolation of the findings to clinical situations since this type of testing can provide valuable insights into fatigue behavior under complex loading conditions. Despite these limitations, the findings provide a valuable basis for future research, reinforcing the idea that varying implant proportions, particularly those smaller than 1:1, offer advantages in implant rehabilitation. Further studies are necessary to elucidate the effect of reduced crown-to-implant ratios on the longevity of rehabilitations and survival rates of implants. This study suggests that a lower crown-to-implant ratio provides favorable outcomes without compromising peri-implant bone health.

## Conclusion

Within the limitations of this in-silico study, the findings suggest that a lower crown-to-implant ratio may be associated with a more favorable stress distribution pattern, potentially minimizing bone overload and contributing to the biomechanical longevity of implant-supported rehabilitations. Although these outcomes provide useful insights into the biomechanical implications of different crown-to-implant ratios, further in vitro and in vivo studies are necessary to confirm these trends and to explore their clinical relevance. Therefore, careful consideration of abutment height and prosthetic dimensions remains essential to reduce biomechanical risks in peri-implant bone tissue.

## Data Availability

The datasets generated during and/or analyzed during the current study are available from the corresponding author on reasonable request.
